# Pathogenic Biallelic Mutations in ECHS1 in a Case with Short-Chain Enoyl-CoA Hydratase (SCEH) Deficiency-Case Report and Literature Review

**DOI:** 10.3390/ijerph19042088

**Published:** 2022-02-13

**Authors:** Carmen Muntean, Florin Tripon, Alina Bogliș, Claudia Bănescu

**Affiliations:** 1 Department of Pediatrics, George Emil Palade University of Medicine, Pharmacy, Science, and Technology of Târgu Mureș, 540142 Târgu Mureș, Romania; 2 Pediatric Clinic, Emergency Clinical County Hospital Târgu Mureș, 540136 Târgu Mureș, Romania; 3 Laboratory of Medical Genetics, Emergency Clinical County Hospital Târgu Mureș, 540136 Târgu Mureș, Romania; florin.tripon@umfst.ro (F.T.); alina.boglis@umfst.ro (A.B.); claudia.banescu@umfst.ro (C.B.); 4 Genetics Laboratory, Center for Advanced Medical and Pharmaceutical Research, George Emil Palade University of Medicine, Pharmacy, Science, and Technology of Târgu Mureș, 540142 Târgu Mureș, Romania

**Keywords:** Leigh syndrome, *ECHS1* gene, neurodevelopment disorder, valine, diet, genotype-phenotype correlations

## Abstract

*ECHS1* gene mutations are known to cause mitochondrial short-chain enoyl-CoA hydratase 1 deficiency, a neurodegenerative disorder characterized by psychomotor development delay, lactic acidosis, and basal ganglia lesions resembling Leigh syndrome. Short-chain enoyl-CoA hydratase 1 (ECHS1) deficiency is a very rare and new disorder, with a wide phenotypic spectrum and different outcomes ranging from neonatal death to survival into adulthood. Since the identification of ECHS1 deficiency in 2014, almost 63 patients with pathogenic mutations in the *ECHS1* gene have been described to date. This paper focuses on the clinical and molecular findings as well as the evolution of a Caucasian girl diagnosed with ECHS1 deficiency who carries a new compound heterozygous mutation in the *ECHS1* gene. Polymorphic symptoms, namely failure to thrive, significant global developmental delay/regression, movement disorders, ocular abnormalities, hearing loss, seizure, and cardiac myopathy, may be a challenge in mitochondrial disorder suspicion. Early diagnosis, an appropriate diet with valine restriction, and trigger avoidance are essential, as there is no effective therapy for the disease. This disorder influences life quality in these patients and their caregivers, and it has the potential to be fatal.

## 1. Introduction

More than 300 genetic forms of mitochondrial disorders have been discovered in the last years due to genome sequencing testing, one of them being short-chain enoyl-CoA hydratase 1(ECHS1) deficiency (MIM #616277). This new disorder recognizes an autosomal recessive inheritance pattern [1]. Biallelic mutations in the ECHS1 gene (encoding short-chain enoyl-CoA hydratase) cause a decrease in ECHS1 activity that manifests with severe neurological impairment, lactic acidosis, and brain Magnetic Resonance Imaging (MRI) abnormalities consistent with Leigh-like syndrome [2,3]. ECHS1, or crotonase, is a 290-amino acid protein placed in the mitochondrial matrix with multiple roles in some metabolic pathways, including the oxidation of fatty acids and the degradation of essential amino acids such as valine (Val) [2,4,5,6]. Short-chain enoyl-CoA hydratase is one of the five enzymes required in the mitochondrial degradation of Val to propionyl-CoA/succinyl-CoA [3,5]. ECHS1 enzyme is involved in the early phases of the Val catabolism pathway. Furthermore, the ECHS1 enzyme has a critical role in the degradation of other branched-chain amino acids, such as tiglyl-CoA (isoleucine pathway), 3-methylcrotonyl-CoA (leucine pathway), as well as crotonyl-CoA (fatty acid oxidation) [7,8]. The ECHS1 enzyme deficiency leads to intermediate metabolite accumulation, such as methacrylyl-CoA, that, after forming compounds with thiol groups, cause reactive intermediate accumulation, such as of S-(2-carboxypropyl)-cysteamine, S-(2-carboxypropyl)-cysteine (SCPC), its carnitine ester (SCPC-C), as well as N-acetyl-S-(2 Carbxypropyl)- cysteine [3,9].

ECHS1 deficiency is a rare mitochondrial disorder. Due to the wide clinical and genetic heterogeneity of mitochondrial disorders, a reliable and fast diagnostic method is necessary [10]. One recent article reported an almost 55% incidence of non-mitochondrial diagnosis within survey participants before their final diagnoses [10]. Because of the wide spectrum and diversity of the symptoms, as well as their lack of specificity at onset and their rarity, combined with the unfamiliarity with the disorder by medical staff, many patients remain undiagnosed or are diagnosed late, as these patients are evaluated by many doctors (mean 8.19, median 5) [10].

Polymorphic clinical features, such as severe developmental delay or regression, feeding difficulties, faltering growth, profound irritability, central hypotonia, seizures, visual impairment, and cardiomyopathy, were previously reported in cases with ECHS1 deficiency [2,4,5,11]. In most cases, a severe phenotype is presented in the neonatal period [12]. The multi-system nature of ECHS1 deficiency and its fluctuating clinical course may evolve in a myriad of ways, making diagnosis and management challenging. Different symptoms and findings may be encountered in patients with the same genotypes [2].

Although significant advances in the molecular basis and genetics of mitochondrial diseases have been made, management involves predominantly supportive care and the early treatment of organ-specific complications, resulting in considerable morbidity and, in those most severely affected, death [12,13]. Clinical manifestations of short-chain enoyl-CoA hydratase (ECHSC) deficiency, therapy, and follow-up are presented in our case that inherited two variants in the *ECHS1* gene, being the first case described in Romania and Eastern Europe.

Our study was endorsed by the board of the Ethics Committee of the George Emil Palade University of Medicine, Pharmacy, Science, and Technology of Târgu Mureș, Romania (approval number 66/27 April 2018). Clinical evaluation and genetic investigation were performed after the informed consent of the child’s parents was obtained.

## 2. Materials and Methods

For genetic analysis, blood samples were collected in EDTA tubes. As mitochondrial disorder was suspected, the multiplex ligation-dependent probe amplification (MLPA) analysis of the mitochondrial DNA extracted from blood sample was performed using SALSA MLPA Probemix P125-C1 Mitochondrial DNA. No deletions or duplications were observed, and neither were point mutations (8993T>G ATP6 gene associated with Leigh syndrome; 3243A>G TL1 gene; 3460G>A ND1 gene; 8344A>G TK gene; 11778G>A ND4 gene; 14484T>C ND6 gene).

Exome sequencing was requested and it was performed from an EDTA blood sample by the Department of Human Genetics Radboud University Medical Center. Briefly, exome enrichment (by using Agilent SureSelectXT Human All exon 50MB), exome sequencing (on the Illumina HiSeq system from Ilumina, Inc., San Diego, CA, USA), read alignment (BWA), and variant calling (by using the Genome Analysis Toolkit GATK (Broad Institute, Cambridge, MA, USA) at BGI-Europe-Denmark) were performed. Copy number variant calling (by using the Copy Number Inference from Exome Reads CoNIFER algorithm), variant annotation, selection, and prioritizing of sequence variants for pathogenicity were performed by applying an-in house strategy developed at the Department of Human Genetics Radboud University Medical Center. Reads were aligned to the human Genome Reference Consortium (GRC) GRCh37/UCSC Genomics Institute hg19.

Moroever, we performed target sequencing for the stored DNA from the patient’s sister after PCR amplification using a BigDye Terminator v3.1 cycle sequencing kit (Thermo Fisher Scientific, Waltham, MA, USA) on an 8-capillary 3500 Genetic Analyzer (Applied Biosystems, Foster City, CA, USA). Data analysis was performed by using Sequencing analyzing v5.4 software. Sequencing analysis revealed the same variants as those observed in the index case, namely c.817A>G (p.Lys273Glu; rs565090080) and c.476A>G (p.Gln159Arg; rs375032130).

## 3. Results

### 3.1. Clinical Summary

#### 3.1.1. Presenting Concerns

The girl was referred to the Pediatric Department from Tîrgu Mureș, Romania, for growth retardation, severe global developmental impairment, epilepsy, and metabolic acidosis with an onset in early infancy (at the age of three months).

The patient, a two-year-old girl, is the second child of healthy, non-consanguineous Caucasian parents. The baby girl was born after an uneventful pregnancy by Cesarean section (C-section) at 41 weeks of gestation with normal birth measurements (weight 3650 g, length 55 cm, and head circumference 35.5 cm) and an APGAR score of 8 at 1 min and 9 at 5 min, respectively. She came from the third pregnancy of her mother. Mild axial hypotonia was noticed at birth. During the first 3 months of her life, she was breastfed, with good gastric tolerance and acceptable weight gain, but with the persistence of hypotonia and no head control, despite physical therapy. At the age of 4 months, she presented irritability, vomiting, and poor weight gain, which became more pronounced when she was switched from breastfeeding to an anti-reflux formula, as the feeding difficulties were initially considered gastroesophageal reflux. At the age of 6 months, more expressed irritability, excessive crying, and a cognitive regress was noticed: she stopped smiling and cooing, and sleep disorders appeared. Facial and limb dystonia, grimacing, and persistent clenched fists were remarked. Abnormal eye movements appeared and were considered as epileptic crisis, so valproic acid therapy was started, but this therapy worsened her symptoms.

The family history revealed a spontaneous abortion of the first fetus and a sister of this girl that died at age 11 months during an acute cardiorespiratory decompensation. The first child (a sister of the described case) was born from a normal pregnancy at 39 weeks of gestation by C-section, with a birth weight of 3500 g, length 54 cm, head circumference 36 cm, and APGAR score of 10. The mother remarked an increase in fetus movements in the last 6 weeks of pregnancy, and fetal monitoring showed tachycardia episodes. In this case, an early onset was present within the 1st day of life with food refusal, marked irritability followed by marked sleepiness, and generalized hypotonia. The newborn, on the 3rd day of life, developed nystagmus, abnormal limb movements, absence of archaic reflexes, bradypnea, and apnea, followed by respiratory distress that required ventilation. Blood gas analysis showed severe metabolic acidosis. An electroencephalogram (EEG) showed an attenuation appearance of delta and theta waves in the frontal area. A cerebral MRI documented marked cerebral atrophy and reduction of the white matter. In evolution, she developed seizures, severe generalized hypotonia, limb dystonia, global developmental delay, growth failure, and loss of gag reflex, so a nasogastric tube was inserted. The evolution was unfavorable, with slow weight gain, severe generalized hypotonia, oral and limb dystonia, and exitus at 11 months old.

#### 3.1.2. Clinical Findings

At the first evaluation in our hospital, the patient presented severe hypotonia and spasticity; she was not fixing or following objects or persons. Additionally, intermittent horizontal and vertical nystagmus, movement disorders like dystonia and hypokinesia; marked irritability, and episodes of inconsolable crying were present. She reacted to voices and noises. No gross dysmorphic features were found during the physical examination. Exercise intolerance was present. She was not able to crawl, sit, or speak.

Gastrointestinal dysmotility was present, and it manifested with early satiety, poor sucking with drooling, vomiting, failure in weight gain, and chronic constipation. 

At that time her anthropometric parameters were: weight 8.75 kg (−2.5SD, <1st percentile); height 82 cm (−1SD, 16th percentile), and head circumference 43 cm (−3SD, <1st percentile), respectively.

### 3.2. Diagnostic Tests

#### 3.2.1. Biologic and Imagistic Assessment

Routine blood tests indicated blood cell count, calcium, phosphorus, alkaline phosphatase, creatine phosphokinase, serum25-hydroxyvitamin D, albumin, glucose, liver enzymes, kidney function, and electrolytes were all within the normal range. Mild metabolic acidosis was noticed. The patient’s metabolic workup is presented in Table 1.

Additionally, the first profile of serum amino acids (by standard tandem mass spectrometry methods) and urinary organic acids analysis (by standard gas chromatography-mass spectrometry) did not point towards the presence of an inborn metabolic disorder.

A second gas chromatographic-mass spectrometric analysis of organic acids in urine as well as plasma amino acids showed slightly to moderately elevated concentrations of some parameters (Table 2). The tiglate metabolites (isoleucine pathway), namely Tiglylglycine, were nondetectable.

Pyruvate dehydrogenase complex activity and cerebral spinal fluid investigations were not performed.

The Nijmegen mitochondrial disease severity score, proposed by Morava et al. and reassessed by Riley, was used, displaying a result of 9, so our patient was classified as having a definite mitochondrial disease [14,15].

The abdominal ultrasound was normal without renal involvement. Echocardiography detected the presence of nonobstructive hypertrophic cardiomyopathy. The ophthalmologic assessment revealed normal ocular fundus, while the visual evoked potential test was abnormal, with no response suggesting cortical blindness. The EEG showed slow activity without epileptic discharges.

The parents did not consent to the muscle biopsy for respiratory chain complexes assessment.

**Figure 1:**
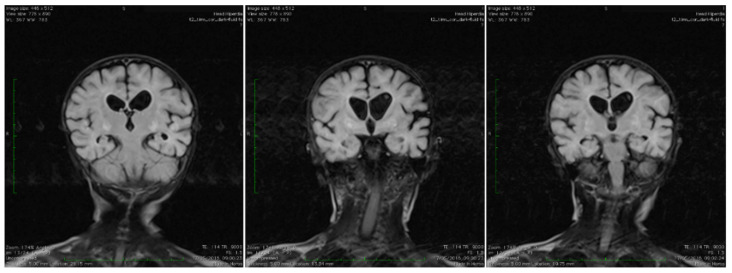
Coronal T2 TIRM dark fluid MRI sequences revealing bilateral basal ganglia hyperintensities and cerebral atrophy.

Brain magnetic resonance imaging (MRI) showed areas with increased T2-signal intensity and inhomogeneous fluid-attenuated inversion recovery (FLAIR) within the basal ganglia as well as cavitation at this level, with extensive diffuse white matter changes in the supratentorial area (leukodystrophy), and brain atrophy with subsequent widening of the perivascular space. Characteristic bilateral abnormal MRI signals in the brain were found (Figure 1).

#### 3.2.2. Genetic Analysis

For genetic analysis, blood samples were collected. Two pathogenic variants in the *ECHS1* gene, one of the genes associated with the mitochondrial disorder (gene panel version DG-2.8) were detected. The index case was compound heterozygous for the c.476A>G (p.Gln159Arg; rs375032130) and c.817A>G (p.Lys273Glu; rs565090080) variants in the *ECHS1* gene, which are pathogenic according to VarSome [16].

Both parents were tested for the *ECHS1* variants identified in the girl. The mother of the index cases was a heterozygous carrier for the c.476A>G (p.Gln159Arg; rs375032130) variant while the father was a heterozygous carrier for the c.817A>G (p.Lys273Glu; rs565090080) variant in the *ECHS1* gene.

Moreover, we performed target sequencing for the stored DNA from the patient’s sister that revealed the same variants as those observed in the index case, namely c.817A>G (p.Lys273Glu; rs565090080) and c.476A>G (p.Gln159Arg; rs375032130).

**Figure 2:**
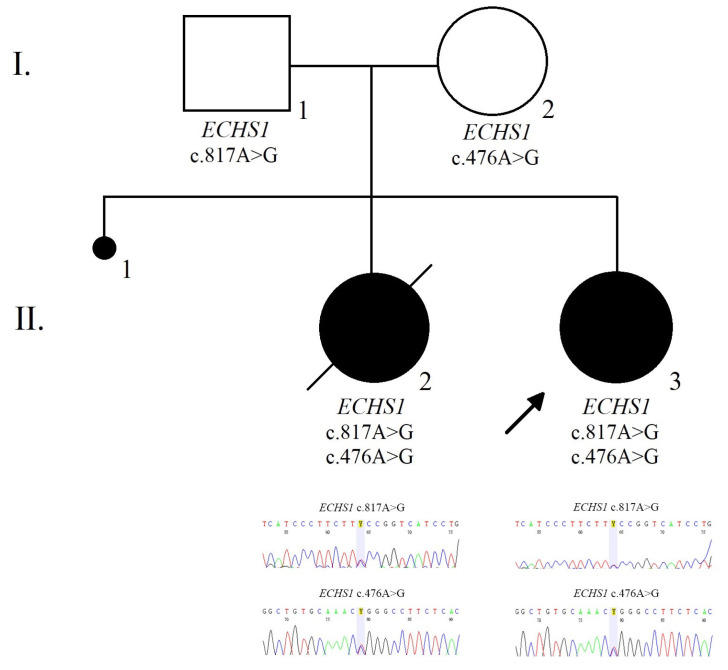
The pedigree and electropherogram illustrate the compound heterozygous mutation in the *ECHS1* gene in the affected child.

The family pedigree illustrates the presence of the *ECHS1* heterozygous mutations within family members (Figure 2).

**Figure 3:**
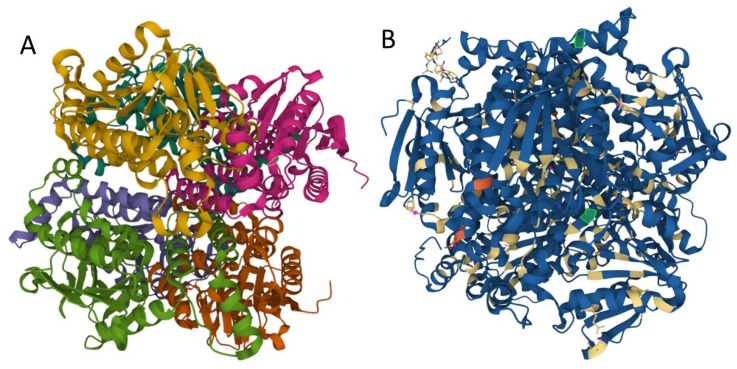
Structure of human enoyl-coenzyme A (CoA) hydratase short chain 1, encoded by *ECHS1* gene. (**A**) the crystal structure at a resolution of 2.55 Å, from the PDB protein databank, structure 2HW5 [18]. (**B**) Missense mutations affect the homohexameric ECHS1 structure. (the location of the changes are represented with green in 2HW5, and were visualized by using Mol * Viewer, a modern web app for 3D visualization and analysis) [19].

Mutations may influence protein folding and stability, function, and protein interactions with other proteins or lipids, etc. We used MutPred2 to model the effects of variants on ECHS1 protein structure and function. MutPred2 is a useful pathogenicity prediction tool, and it assigns putative molecular alterations [17]. For ECHS1 p.Gln159Arg (Q159R) substitution, MutPred2 predicted loss of relative solvent accessibility, loss of pyrrolidone carboxylic acid, and altered transmembrane protein, while for p.Lys273Glu (K273E) it predicted loss of ubiquitylation and methylation at K273, altered Coiled coil, and loss of Helix (Figure 3).

#### 3.2.3. Prenatal Testing and Genetic Diagnosis

Since the *ECHS1* pathogenic variants have been identified in family members, genetic counseling was offered. The recurrence risk is 25%, and prenatal testing in a future pregnancy and preimplantation genetic testing is recommended [12].

### 3.3. Therapeutic Focus and Assessment

Until the results of genetic tests were available, different therapies were proposed, but without results. Before metabolic evaluation, levetiracetam was administrated, but the symptoms worsened, so it was stopped. Different therapeutic cocktails with thiamine 100 mg daily, followed by carnitine (50 mg/kg/day) and folinic acid (1 mg/kg/day), and coenzyme Q10 were considered without any improvement.

Because of swallowing difficulties and significant failure to thrive at 2.5 years of age, a percutaneous endoscopic gastrostomy (PEG) tube was inserted, which led to some weight gain. Furthermore, a mild reduction in valine intake was recommended, so valine serum levels were 140–160 μmol/L. The last recommended treatment was L-arginine on the oral route, while the intravenous route was proposed in case of acute illness. With this treatment, the lactate level decreased from 3.88 to ≤2.6–2.1 mmol/L and the symptoms improved. Additionally, a low valine diet was started, avoiding any amino acid deficiencies and meeting energy needs (~80 kcal/kg/day). Valine intake was reduced significantly (total protein intake 2 g/day, valine dietary intake ~35 mg/kg/day) with close monitoring of the patient’s valine levels and the other essential amino acids.

### 3.4. Follow-Up and Outcome

A transient increase of plasma lactate was found during infections. During mitochondrial cocktail therapy, the patient did not have any control of her head and did not sit. She stopped smiling or crying. She reacted neither to visual or auditory stimuli and severe hypotonia was present. Dystonia and nystagmus were present.

**Figure 4:**
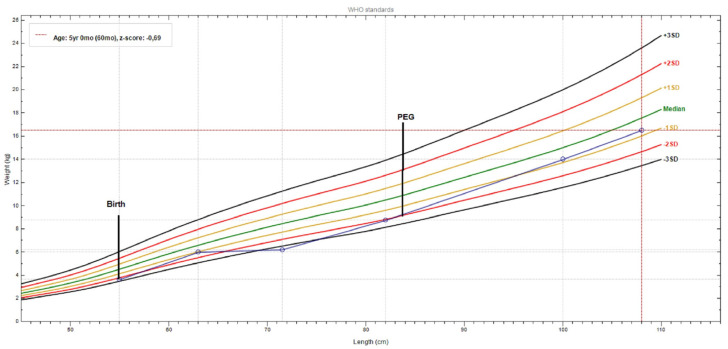
Patient weight-for-length chart, from birth to present. The growth curve reveals a significant weight gain after PEG (percutaneous endoscopic gastrostomy) tube placement and a valine-restricted diet. (chart realized with WHO Anthro v3.2.2 software).

Until PEG tube placement, weight gain was severely compromised (−2.5SD); after PEG insertion plus mild valine restriction (at 2.5 years), the weight gain started to ameliorate, and when strict valine restriction was started this was more evident (Figure 4).

A remarkable improvement in her symptoms (dystonia, muscle spasms) and neuromotor development was obtained in one year with a strict valine-restricted diet, started at 5 years. She started to hold her neck, sit with support, and grasp a toy, but she was not able to pick it up. She started to coo, to react to voices and noise, and to smile; she turned her head towards a sound and tracked an object. These were achieved significantly by reducing the valine serum levels from 162 to 77 μmol/L (0.91 mg/dL) (reference range 1.1–3.3 mg/dL).

## 4. Discussion

Mitochondrial diseases are under-diagnosed disorders because of their rarity. The process of obtaining a correct diagnosis is long and may take more than one year. According to Grier et al., a non-mitochondrial diagnosis was made in half of the cases before their final diagnoses [10]. This was due to the wide spectrum and diversity of the symptoms and the disease’s rarity [2,13]. A total of 63 ECHS1 deficiency patients aged between 0 and 18 years have been described to date.

After a long period, even if mitochondrial diseases are finally successfully diagnosed, treatment options are limited, and gene therapy is under research [13]. However, the correct diagnosis permits optimal management and a reduction in evolution rate.

In our case, the mitochondrial disease diagnosis was in accordance with data published in a recent study [10].

Mitochondrial disease cases frequently have a multisystem involvement: neurological, muscular, ophthalmological, cardiac, kidney, liver, gastrointestinal tract, vision, hearing, endocrine, and growth [20], so a high index of suspicion is required in such cases.

Since no clinical diagnostic criteria for ECHS1 deficiency are available, neonatologists, as well as pediatricians should perform a periodic reevaluation of patients with developmental delay, hypotonia, ophthalmologic anomalies, gastrointestinal dysmotility, and failure to thrive without genetic confirmation. Moreover, serum lactate and an ammonia level should be evaluated, especially when the serum amino acids and urinary organic acids profiles are in the normal range [2,14,15]. These children experience regression of language and motor skills, muscle weakness, hearing loss, seizures, developmental delay, and growth failure [14,15,21].

A very recent article proved the utility of genome sequencing for diagnosis in a pediatric cohort with suspected mitochondrial disease [14], and the authors considered that this is the best method to achieve accurate diagnosis in 55% of investigated cases and to avoid invasive and dangerous investigation, namely muscle biopsy. Muscle biopsy for respiratory chain complex assessment was not performed in our case, and the diagnosis was established by exome sequencing. Because of the severity of the multisystem involvement, our patient had the high-risk patient group criteria, so this invasive procedure requiring general anesthesia was not performed. In such cases, a mitochondrial disease score followed by genetic testing should be performed before indicating a muscle biopsy [15]. According to Parikh et al., a muscle biopsy should be performed when the diagnosis cannot be confirmed by DNA testing [21]. Pathogenic variants in nuclear-encoded genes are proven in 75% of pediatric mitochondrial diseases [14].

Suspicion of having a mitochondrial disorder was raised after the serum lactate and pyruvate were measured. A high lactate-to-pyruvate (L/P) ratio is relevant and supportive for mitochondrial disease diagnosis [15]. The lactate level is influenced by other factors: inadequate collection technique, sepsis, circulatory collapse, hypoxia, hypotension, organ perfusion deficiency, seizures due to hypoxic brain injury, poisoning, systemic disease, thiamine deficiency, or secondary mitochondrial dysfunction [20].

The blood lactate-to-pyruvate (L/P) ratio is used to distinguish between pyruvate dehydrogenase complex deficiency and other causes of inborn lactic acidosis. Respiratory chain defects or tricarboxylic acid cycle disorders usually result in an elevated lactate level in conjunction with L/P ratios > 25 [20]. Increased lactate levels may be found just in the acute phase in individuals with heterozygous mutations in the *ECHS1* gene: c.5C>T (p.Ala2Val) and c.176A>G (p.Asn59Ser) [2].

Higher transitory ammonia was found, but initial chromatography of plasma amino acids and organic acids did not reveal pathological changes.

Several reports regarding ECHS1 deficiency reported secondary functional pyruvate dehydrogenase complex (PDC) deficiency, which was not investigated in our case as the L/P ratio was >25 mmol/L [22].

A recent report discussed the importance of assessing lactate and pyruvate in urine and plasma samples, as a discrepancy between their results was observed [2]. Recent articles support the idea that detecting the significant value of urine 2-methyl-2,3-dihydroxybutyric acid is specific for primary deficiency of ECHS1 [6,11], but its level is directly related to lactic acidosis. This may be the explanation for the initially normal and later slightly increased values in our case. This supports the recommendation to collect biological samples during a decompensation episode [11]. A more recent study stated that this metabolite is a nonspecific biochemical finding [5]. An increased level of 2-methyl-2,3-dihydroxybutyric was detected in our patient, similar to the report of Peters et al. which suggested this metabolite undergoes a common biochemical change in ECHS1 [23].

Accumulation of methacrylyl-CoA (also known as methacryloyl-CoA), a toxic intermediate metabolite in the valine catabolic pathway, was hypothesized to represent the central pathophysiological mechanisms of ECHS1 deficiency [1,2].

Because of the characteristic pattern, a cranial MRI is recommended to be performed in such cases, as it can be itself diagnostic [2,15]. Moreover, conjugate eye deviation, hypotonia, and brain lesions in the basal ganglia and hyperlactatemia, may be found [2]. A characteristic brain MRI scan image exhibits bilateral and symmetrical T2 hyperintensity of the caudate nucleus, putamen, and globus pallidus, findings consistent with Leigh syndrome [2]. Similar findings were described in our case.

Another challenge for diagnosis is the fact that the phenotype of the patients depends on the pathogenicity of the variants identified in heterozygous compounds. Severe symptomatology was noticed in biallelic mutations in the *ECHS1* gene [4,6].

Interestingly, similar to our case, these children were full-term or late-term babies [4,24].

In the case series reported by Haack et al., early onset of symptomatology was noticed in 7 of 10 patients, while cardiac involvement was noticed just in those with severe evolution and early fatality (40% cases), but only in one case with c.476A>G mutation (c.(176A>G);(476A>G) p.(Asn59Ser); (Gln159Arg)) [4]. Our case was a compound heterozygous c.817A>G (p.Lys273Glu; rs565090080) and c.476A>G (p.Gln159Arg; rs375032130), carrying the c.476A>G mutation reported previously by Haak et al. [4].

According to Ensembl genome database, the minor allele frequency of *ECHS1* c.476A>G (rs375032130) and c.817A>G (rs565090080) is less than 0.01 [25]. Moreover, the Exome Aggregation Consortium (ExAC) reported extremely rare variants of the *ECHS1* gene in the general population and individuals were identified only in a heterozygous state, as follows: 7 (c.817A>G) and 30 (c.476A>G) of 282,372 unrelated individuals [26].

In a recent article that evaluated ECHS1 cases, these compound variants were not described in any case [1]. The two mutations described here have already been described in other patients, although not associated together. According to recent articles and the VarSome database, both of these variants c.476A>G (p.Gln159Arg) and c.817A>G (p.Lys273Glu) are pathogenic [5,14,16].

Both siblings were found as compound heterozygotes for a c.817A>G (p.Lys273Glu) and a c.476A>G (p.Gln159Arg) missense mutation in the *ECHS1* gene. Both girls presented the clinical features of an ECHS1-related disorder.

The homozygous state for c.817A>G (p.Lys273Glu) resulted in early-onset and premature death [7], compared to the homozygous state for c.476A>G (p.Gln159Arg), which is associated with a longer survival during childhood (few years). On the other hand, the compound heterozygous state c.244G>T (p.Val82Leu), c.476A>G (p.Gln159Arg) and c.229G>C (p.Glu77Gln), c.476A>G (p.Glu159Arg) [4] were associated with survival into adulthood.

Cardiac involvement, namely hypertrophic cardiomyopathy, was previously reported by Haack et al. in the heterozygous compound for the c.476A>G variant [4]. Our case, a heterozygous compound for the c.476A>G variant, presented a similar heart disorder.

The overlapping clinical and biological features observed in our case were in patients c.476A>G. No gender preference was observed for the c.476A>G (p.Gln159Arg) and c.817A>G(p.Lys273Glu) variants [4,27]. A recent paper reported a 7-month-old infant with ECHS1 deficiency presenting with hypotonia, conjugate deviation, and severe ketoacidosis, with normal lactate levels. This infant was proven to have heterozygous mutations in ECHS1: c.5C>T (p.Ala2Val) and c.176A>G (p.Asn59Ser), which are common in Japanese patients with ECHS1 deficiency [2].

We explored the PubMed database for compound heterozygous patients with ECHS1 deficiency carrying at least one variant, as in the described case. We did not find another reported case with similarly combined alleles as our case. All cases are described in Table 3 [4,27,28,29,30,31,32].

Unfortunately, the prognosis is reserved as the disease results in further progressive deterioration, and there is no medical therapy for ECHS1D, with gene therapy being under research at the time of writing.

Physical exercise and physical therapy are proven approaches for improving mitochondrial function, but a therapist or a kinesiologist should supervise them [33].

Some drugs, namely valproic acid, topiramate, vigabatrin, statins, aminoglycoside and erythromycin, and acetaminophen, should be avoided in these cases, or should administered under close monitoring [12,33]. ECHS1 deficiency symptoms may be exacerbated during illnesses such as viral infections, as happened in our case.

Preventive measures or rapid treatment should be initiated to avoid fast systemic decompensation during a minor or more severe condition like vomiting, dehydration, fever, anesthesia, surgery, or prolonged fasting. Hospital admission is recommended where the patient should be provided with 5–10% dextrose-containing intravenous fluid, correction of any electrolytes and metabolic abnormality, as well as stopping exposure to potentially toxic medications. Lactated Ringer’s solution should not be used in mitochondrial patients, while lipids can be used when necessary. Initially parenteral, then enteral feeding should be considered [20,33,34].

Immunizations should be offered to prevent infectious diseases in these patients [33].

Symptomatic therapies represent the existing clinical management for mitochondrial disorders. They address the patient’s manifestations (namely anti-epileptic drugs or a ketogenic diet, β-blockers, renal replacement therapy, cochlear implants, organ transplantation, etc.) [13], but their efficiency is debatable. Moreover, vitamin cocktails are useless [35]. A ketogenic diet, prolonged propofol infusions, and valproic acid should be avoided [12].

Early therapeutic interventions are of great importance to prevent or reduce neurological damage, especially in cases with early diagnosis and/or mild clinical symptoms [3,13]. A recent report showed that a valine-restricted diet could be prescribed with promising results for clinical improvement, but the data were influenced by the short-term follow-up of this case [36]. A newer study reports the long-term results in the clinical, neuroimaging, and biochemical responses and clinical improvement in three children with ECHS1 deficiency that were treated with a valine-restricted diet and close monitoring of plasma valine levels [3].

The target range for serum valine levels (74–90 μmol/L) was noticed in our case within the first two months of protein-restricted diet, in agreement with those proposed by Abdenur et al. [3]. At the time, no isoleucine supplementation was necessary, as isoleucine and leucine levels were within the normal range, showing results similar to those described by Abdenur et al. [3]. Recently, Kuwajima et al. reported that protein restriction therapy may alleviate the symptoms of ECHS1 deficiency [37]. A low-protein diet may prevent disease progression, while a valine-restricted diet may lead to a clinical and neuroradiological improvement in ECHS1 deficiency [38].

N-acetylcysteine supplementation proved to be efficient for children with ECHS1 deficiency [1]. A new proposed therapy is represented by Triheptanoin (7 carbon fatty acid), which has already been used with variable results in intractable epilepsy in children and adults [39,40]. Triheptanoin, an anaplerotic treatment, increases energy generation via the tricarboxylic acid cycle or the Krebs cycle [35,36]. Triheptanoin directly enters mitochondria as a C7 fatty acid, which is metabolized to one C3 Propionyl CoA and two C2 Acetyl CoA. The latter enters the Krebs cycle directly, while one C3 Propionyl CoA is converted to methylmalonyl-CoA and then to succinyl-CoA, a Krebs cycle intermediate [35,39]. Triheptanoin is a medium-chain triglyceride with 3 odd fatty acids, tasteless, and well-tolerated in ascending doses up to 100 mL/day (up to 40% of daily caloric intake), with mild gastrointestinal side-effects [39], and may represent a treatment option in ECHS1 deficiency [35].

Quality of life in these patients and their caregivers are influenced by progressive evolution. Prognosis is poor, with a short survival rate of about 70 months, but cases with neonatal deaths or adulthood survival up to 32 years have also been reported [41,42].

The particularity of our case comes from the presence of a compound heterozygous *ECHS1* gene by the presence of c.476A>G (p.Gln159Arg; rs375032130) and c.817A>G (p.Lys273Glu; rs565090080) variants. The *ECHS1* gene is responsible for ECHS1 deficiency, a new disorder, with an autosomal recessive inheritance pattern. The patient presented a severe phenotype but showed remarkable improvement in her symptoms (dystonia, muscle spasms) and neuromotor development during the valine-restricted diet.

## 5. Conclusions

The ECHS1 c.817A>G (p.Lys273Glu; rs565090080) and c.476A>G (p.Gln159Arg; rs375032130) variants in a compound heterozygous state lead to early symptom occurrence. A neonate or infant with growth retardation, developmental delay or arrest, and lactic acidosis should raise the suspicion of a mitochondrial disorder. Early recognition is essential. ECHS1 deficiency may represent a challenging diagnosis in the case of normal amino and organic acid profiles. The results of a valine-restricted diet and Triheptanoin are encouraging, representing a promising therapy until gene therapy becomes available.

## Figures and Tables

**Table 1:** Metabolic profile.

Serum Parameters	Measured Value	Normal Range
lactate	43.07 mmol/L	<2.5 mmol/L
ammonia	1238 μg/L	187–869 μg/L
lactic acid	3.88 mmol/L	0.5–2.2 mmol/L
pyruvate	0.7 mmol/L	0.41–0.67 mmol/L

**Table 2:** Serum amino acid and urinary organic acid profile.

Plasma Amino Acid	Value	Urinary Organic Acid	Value
Cystine	↑	3-hydroxyisovaleric acid	↑↑
Valine	↑	pyruvic acid	↑↑
Alpha alanine	N	2-methyl-2,3-dihydroxybutyric	↑
Acyl-carnitine	N		

N: normal value; ↑: increased value, ↑↑: two times normal value.

**Table 3:** Phenotypic, metabolic, and MRI spectrum in children with a mutation in ECHS1 in the heterozygous state (c.476A>G (p.Gln159Arg; rs375032130) or c.817A>G (p.Lys273Glu; rs565090080).

Patient	1	2	3	4	5	6	7	8	9	10	11
Ethnicity	Romanian parents	Iraqi-Turkish Jewish/mother Iraqi -Libyan Jewish	Japanese and American parents	Netherlands parents	German parents	French-Canadian parents	NL	NL	Italian parents	Belgian parents	Greek parents
Genetic mutation	c.476A>G/c.817A>G	c.433C>T/c.476A>G	c.176A>G/ c.476A>G	c.161G>A/c.817A>G	c.229G>C/c.476A>G	c.538A>C/c.476A>G	c.518C>T/c.817A>G	c.476A>G/c.538A>G;	c.476A>G/c.139G>A	c.239C>T/c.817A>C	c.476A>C/ c.542G>T
Protein effect	p.Gln159Arg/p.Lys273Glu	p.Leu145Phe/ p.Gln159Arg	p.Asn59Ser/ p.Gln159Arg	p.Arg54His/ p.Lys273Glu	p.Glu77Gln/ p.Gln159Arg	p.Thr180Ala/p.Gln159Arg	p.Ala173Val/ p.Lys273Glu	p.Gln159Arg/ p.Thr180Ala	p.Gln159Arg/p.Val47Met	p.Pro80Leu/p.Lys273Glu	p.Gln159Arg/p.Arg181Leu
Allele source	c.476A>G mother c.817A>G father	NL	c.476A>G mother c.176A>G father	ND	c.476A>G mother father- ND	c.538A>C mother father NA	c.518C>T father c.817A>G mother	Parents- heterozygous for variants	c.476A > G father c.139G > A mother	NL	NL
Sibling (sex, mutation)	F, dead/c.476A>G/c.817A>G	No	F, dead in 1st day of life: respiratory failure, severe lactic acidosis; mutation NL	NL	M, alive, c.229G>C/= (p.Glu77Gln/=) (heterozygous carrier)	NL	NL	NL	NL	NL	NL
Sex	F	F	F	M	F	F	M	F	M	M	F
Age of onset	3 mo	early infancy	birth	birth	11 mo	1 mo	8 ys	17 mo	birth	35 mo	8 mo
First neurological signs/symptoms	hypotonia	hypotonia	hypotonia	hypotonia	fist-clenching, teeth-gnashing, horizontal nystagmus	development delay or regression	dyskinesias		hypotonia, lower limbs-flexed on the trunk	lower limb paroxysmal dystonia	encephalopathy
Neurologic involvement/characteristics	microcephaly, hypotonia dystonia, spasticity, nystagmus, seizure, inconsolable crying, optic atrophy, global developmental delay, hearing loss?	microcephaly, hypotonia, global developmental delay, optic atrophy, hearing loss	deafness	microcephaly, hypotonia, dystonia, spasticity, inconsolable crying, global developmental regress	dystonia, developmental delay, spastic, nystagmus, hearing loss, optic atrophy	microcephaly, hypotonia, dystonia, nystagmus, optic atrophy, hearing loss	dyskinesias, dystonia	hypotonia, dystonia global developmental delay	hypotonia, limbs spasticity, hearing loss	dystonia, spasticity, mild developmental delay	microcephaly, nystagmus, severe development delay optic atrophy, hearing loss
Seizure, age of onset	Yes, 6 mo	No	Yes	Yes, 1.3 ys	Yes	Yes / +	No	No	No	No	No
Feeding difficulties	Yes	Yes	NL	yes	NL	NL	No	NL	NL	No	Yes
Cardiomyopathy/Cardiac involvement	HCM	transient hypertrophy of the interventricular septum	HCM, cardiac failure	No	No	NL	NL	No	LVH	No	No
Outcome alive/death (age)	Alive 6 ys	Alive at 7 ys	Death at 4 mo	Death at 7.5 ys	Alive at 31 ys	Alive at 12 ys	Alive at 8 ys	Alive at 4.5 ys	Death at 62 d	Alive 6 ys	Death 9 ys
Metabolic workup											
Plasma Lactate	High	High	High	ND	High	N	N	High	High	N	High
Plasma Pyruvate	High	NL	NL	NL	N	NL	N	N			
Alanine	N	High	NL	NL	NL	NL	N	NL	High	N	N
Urinary organic acids	NL	NL	metabolic profiling (amino acid analysis, urine organic acid analysis, acylcarnitine analysis) was unremarkable	NL	NL	severe ketosis and hyperlactaturia (resolved with treatment)	N	normal plasma amino acids, urine organic, and amino acid analysis	NL	NL	NL
2-methyl-2,3-dihydroxybutyric acid	High	High	N	NL	N	NL	N	NL	High	N	N
MRI changes	brain atrophy, hyperintensity within putamen, cavitation at this level, extensive diffuse white matter changes	atrophy of the cerebellum and brain stem, mild ventricular dilatation, generalized atrophy of the grey matter, thinning of the corpus callosum	moderate brain atrophy, low intensity in cerebral white matter	Extensive brain atrophy, widening of the subarachnoid space and of the ventricular system	no atrophy, hyperintensity in nucleus caudatus and putamen	cerebellar atrophy, hyperintense T2-weighted images in the putamen, globus palidus, caudate nuclei	regions of increased T2 and FLAIR signal and of hypointense T1 signal in the globus pallidus bilaterally with mild diffusion restriction	globus pallidus, putamen, caudate nuclei, basal ganglia T2 hyperintensity	asymmetric ventricular dilatation, partial agenesis of the posterior part of the corpus callosum, basal ganglia, a slight increase of T2 WM signal intensity, germinal cyst in the thalamo-caudate notch	asymmetric cavitation of globus pallidus, bilateral T2-WI hyperintensity, restricted diffusion	globus pallidus, caudate nuclei T2 hyperintensity
References	Our case	[27]	[4]	[4]	[4]	[28]	[29]	[30]	[31]	[32]	[32]

d: day; mo: months; ys: years. M: male; F: female; HCM: hypertrophic cardiomyopathy; LVH: left ventricular hypertrophy; NL: not listed; N: normal; WM: white matter.

## Data Availability

Data and materials are available from the corresponding author on reasonable request.
